# A Fibromatosis Case Mimicking Abdominal Aorta Aneurysm

**DOI:** 10.1155/2013/124235

**Published:** 2013-07-16

**Authors:** Arzu Tasdemir, Cemal Kahraman, Kutay Tasdemir, Ertugrul Mavili

**Affiliations:** ^1^Department of Pathology, Educational and Research Hospital, Sanayi Mah., Atatürk Bulvarı Hastane Cad., Kocasinan, 38010 Kayseri, Turkey; ^2^Department of Cardiovascular Surgery, Erciyes University Medical School, Talas Yolu 5. Km, 38039 Kayseri, Turkey; ^3^Department of Radiology, Erciyes University Medical School, Talas Yolu 5. Km, 38039 Kayseri, Turkey

## Abstract

Retroperitoneal fibrosis is a rare fibrosing reactive process that may be confused with mesenteric fibromatosis. Abdominal aorta aneurysm is rare too and mostly develops secondary to Behcet's disease, trauma, and infection or connective tissue diseases. Incidence of aneurysms occurring as a result of atherosclerotic changes increases in postmenopausal period. Diagnosis can be established with arteriography, tomography, or magnetic resonance imaging associated with clinical findings. Tumors and cysts should be considered in differential diagnosis. Abdominal ultrasound and contrast-enhanced computerized tomography revealed an infrarenal abdominal aorta aneurysm in a 41-year-old woman, but, on surgery, retroperitoneal fibrosis surrounding the aorta was detected. We present this interesting case because retroperitoneal fibrosis encircling the abdominal aorta can mimic abdominal aorta aneurysm radiologically.

## 1. Introduction

Retroperitoneal fibrosis is a rare reactive process. It is two to three times more common in men, and most patients present with it during the fifth or sixth decade of life [1]. It is unusual before the age of 20 or over the age of 70. It is characterized by diffuse or localized fibroblastic proliferation in the retroperitoneum causing compression or obstruction of the ureters, aorta, or other vascular structures.

Abdominal aorta aneurysms (AAAs) are vascular pathologies that mostly develop in advanced-age group due to atherosclerosis. AAAs have a male predominance. But following menopause, female/male ratio becomes equal. Aneurysms become symptomatic after they reach a certain diameter. The diagnosis is established radiologically. But sometimes, benign or malignant lesions may mimic AAAs by surrounding the aorta. Herein, we report a 41-year-old woman who had complaints of abdominal and back pain and was diagnosed as AAA on CT and USG. But on surgery, it was seen that the periaortic thickening was due to retroperitoneal fibrosis. Therefore, we aimed to present this rare case and stress that retroperitoneal fibromatosis should be kept in mind in the differential diagnosis of AAA.

## 2. Case Report

**Figure 1:**
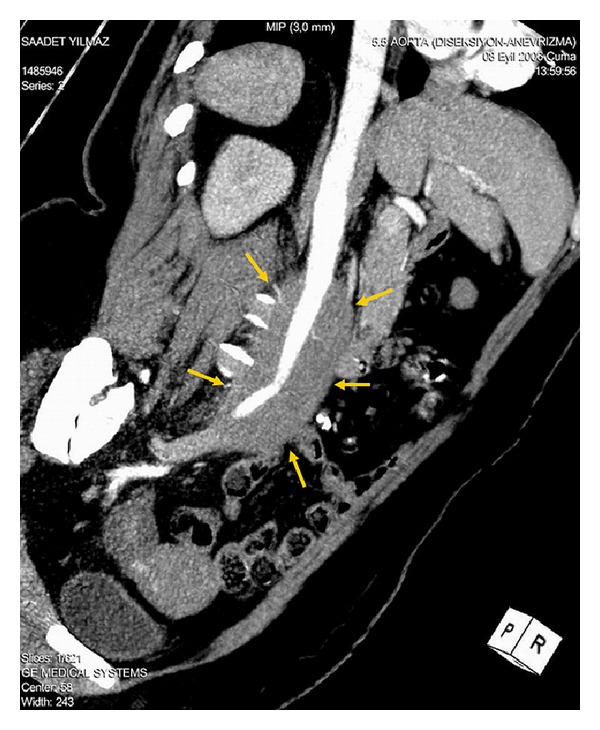
Preoperative contrast-enhanced axial CT images demonstrate that the aorta is surrounded by a soft-tissue mass (yellow arrows).

A 41-year-old woman was admitted to the cardiology clinic because of abdominal and back pain. Physical examination revealed a mass in the subxyphoideal region. An abdominal aorta aneurysm with thrombosis was detected on abdominal ultrasound. CT was obtained for showing the extent of the lesion. CT showed an infrarenal aneurysm (Figure 1) that extends through terminal aorta under renal arteries and reaches to a diameter of 6 cm in some areas. Blood pressure was 120/70 mmHg, and pulse was regular with a rate of 88 bpm. Other organ systems were normal. Laboratory findings of the patient were as follows: hemoglobin: 13,5 g/dL, WBC: 8000 cell/mm^3^, hematocrit: 42.4%, glucose: 96 mg/dL, and BUN: 17 mg/dL. Her family history was unremarkable.

**Figure 2:**
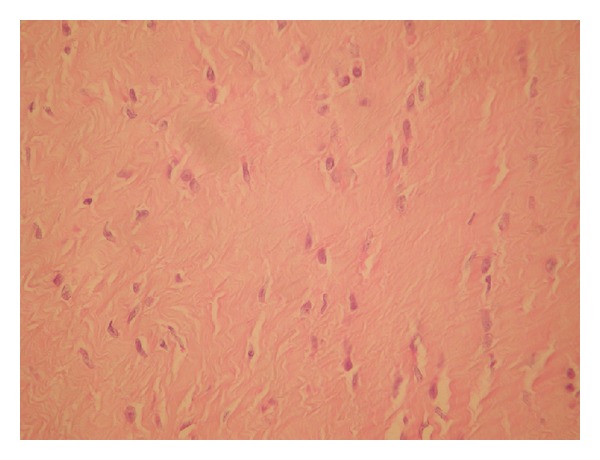
Pathological examination shows fibromatosis (Hematoxylin-Eosin, 40x).

Surgical operation was planned. A midline incision was made to enter abdomen. Intra-abdominal organs were closed by towel dressings to provide the exposure needed. A mass encircling the aorta was encountered. Posterior peritoneum and neighboring structures were firmly adhered to the mass. Complete resection was impossible because the mass was surrounding the aorta and neighboring structures. A diagnostic biopsy was taken from the mass. Intraoperative pathological evaluation of the specimen was considered as fibromatosis, and the operation was terminated. Histopathology of the specimen was also reported as fibromatosis (Figure 2). The patient was referred to our oncology department. Adjuvant chemotherapy was recommended. The patient refused the treatment, so she was discharged on day 7 after operation and was followed up as outpatient. No enlargement was detected in the mass on control CT performed 6 months later.

## 3. Discussion

Retroperitoneal fibrosis is quite uncommon, with an incidence estimated to be less than 1 per 10.000 patients [2]. It is characterized by diffuse or localized fibroblastic proliferation and a chronic lymphoplasmacytic infiltrate in the retroperitoneum causing compression or obstruction of the ureters, aorta, or other vascular structures. Although described years earlier, it was not until the publication in the English literature by Ormond in 1948 that this disease became more widely recognized [3]. Approximately 5%–25% of AAAs is associated with perianeurysmal fibrosis, which likely represents an early or mild form of retroperitoneal fibrosis.

Radiologic imaging methods such as ultrasound, computerized tomography, magnetic resonance imaging, and arteriography decreased the probability of misdiagnosis of vascular pathologies. But still misdiagnosis may occur. Fibromatosis surrounding the aorta is one of the most important pathologies that should be included in the differential diagnosis of AAA cases. In our case, retroperitoneal fibromatosis was mimicking abdominal aorta aneurysm in localization, and symptoms and abdominal CT and ultrasonographic appearance were consistent with abdominal aneurysm. However, when we think retrospectively, being 41 years old, having no exact etiologic cause, and having a homogenous appearance all around could be important clues to exclude aorta aneurysm. Arteriography can provide important information in assessing such equivocal conditions.

Retroperitoneal fibromatosis is a rare reactive-benign process [1]. It grows out of muscular connective tissue. The real nature of fibromatosis is not clear. But cytogenetic or molecular genetic techniques enhance the probability of clonal neoplastic proliferation [4]. Although the etiology of fibromatosis is not clear enough, trauma, radiation, sex hormones, or hereditary defects can be responsible. Besides, it can develop on a surgical scar tissue or it can accompany pregnancy [5]. Some authors reported retroperitoneal fibromatosis in patients with familial adenomatous polyposis (Gardner's syndrome) [4]. Solitary form of fibromatosis had been rarely reported [5]. Our patient did not have any history of trauma or intra-abdominal/retroperitoneal infection.

Fibromatosis is divided into two major groups: superficial and profound. Superficial forms (palmar, plantar, juvenile, and aponeurotic fibromatosis) typically have small volumes and grow up slowly [4, 5]. Ninety-eight percent of all desmoid tumors is superficial and 2% is profound. Profound fibromatosis is located at intra-abdominal, retroperitoneal, or mesenteric regions. They exhibit aggressive features and may have recurrences.

Most of the patients are asymptomatic until the mass reaches great volumes. Depending on localization of intestinal obstruction, abdominal pain, gastrointestinal bleeding, or fever unknown origin can be seen. Symptomatic patients present with vague, nonspecific abdominal symptoms, including dull, poorly localized back or flank pain. In this case, retroperitoneal fibromatosis caused back pain.

Despite the great probability of recurrence, surgery takes great part in treatment of fibromatosis. Complete resection has been suggested as a first choice, and if not, mass reduction has been suggested. Large excision of tumor decreases recurrence risk [4]. However, conservative surgical procedure has been considered in conditions where vital organs have been involved. In these cases, adjuvant chemotherapy (radiotherapy, NSAI treatment, antiestrogen or antineoplastic agents, or combination of them) can be applied. In our case only diagnostic exploration could be done because aorta and bilateral common iliac arteries were encircled by mass. There was no growth in fibromatosis on the 6th month followup.

*In conclusion,* it is necessary to consider fibromatosis with retroperitoneal localization that was found to be rare in differential diagnosis of abdominal aorta aneurysm. Abdominal MRI and/or CT before surgical procedure can provide important clues to exclude abdominal aorta aneurysm.
